# Impact of motion limits on sloped wave energy converter optimization

**DOI:** 10.1098/rspa.2015.0768

**Published:** 2016-03

**Authors:** Rémy Pascal, Grégory S. Payne

**Affiliations:** 1 Abengoa Seapower, Edinburgh EH9 3FB, UK; 2 Institute for Energy Systems, University of Edinburgh, Edinburgh EH9 3FB, UK

**Keywords:** wave energy, potential flow, optimization

## 1. Introduction

In a previous article [1] (subsequently referred to as the ‘original study’ and whose prior reading is recommended to make the most of what follows), the authors explored the concept of sloped power take-off (PTO) for a free-floating wave energy converter (WEC) using linear potential flow theory. Part of the study focused on the optimization of four parameters: the mass reference *m*_2_, its vertical position *w*_G_2___*r*_, the PTO angle *θ*_0_ and the magnitude of the linear damping *α*.

It was decided for the optimization part of the original study to exclude configurations exhibiting normalized motion amplitude (NMA) maxima in surge, heave and pitch above a certain limit, or threshold. This method to keep results realistic within the context of linear potential flow theory was chosen over adding extra damping coefficients to the hydrodynamic model. The reasoning is that, as the PTO angle varies between configurations, the PTO provides more or less damping in pitch for the same *α*. Therefore, some configurations require less additional hydrodynamic damping (representing shape drag) than others to keep pitch normalized motion amplitudes within a realistic limit. Adding a fixed additional damping in pitch would dissipate energy, and therefore penalize some configurations more than others.

In the original study (§4b(i)), a normalized motion amplitude threshold of 10 was used. Experimental normalized motion amplitudes of this order of magnitude have been reported in the literature [2, p. 44]. Using this value effectively reduced the experimental plan of the initial study from 4000 randomly generated configurations to around 600. No configuration with PTO angle $≳-30^{\circ }$ (i.e. close to vertical) was selected with this approach. One could argue that point absorbers with vertical PTO do exist [3] and that they were not duly considered. The argument was that self-referenced heaving point absorbers with vertical PTO are intrinsically unstable in pitch in most cases [4] and that the optimization method adopted was in fact inherently favouring the higher pitch stability provided by a sloped PTO. In any case, it was acknowledged that the simulation method used had its limitations, but that no ideal mitigation strategy for it was available when considering large numbers of configurations with varying hydrodynamic characteristics.

During the review process, one of the reviewers duly questioned the selection of 10 as a normalized motion amplitude threshold. The issue was acknowledged by the authors but addressing it was beyond the scope of the original study and this brief report aims therefore to investigate it further.

While retaining the simulation method and the principle of excluding WEC configurations based on motion threshold, the evolution of the parameters’ optima as a function of the selected thresholds is presented. The validity of the overall method based on these new observations is discussed.

**Figure 1.**
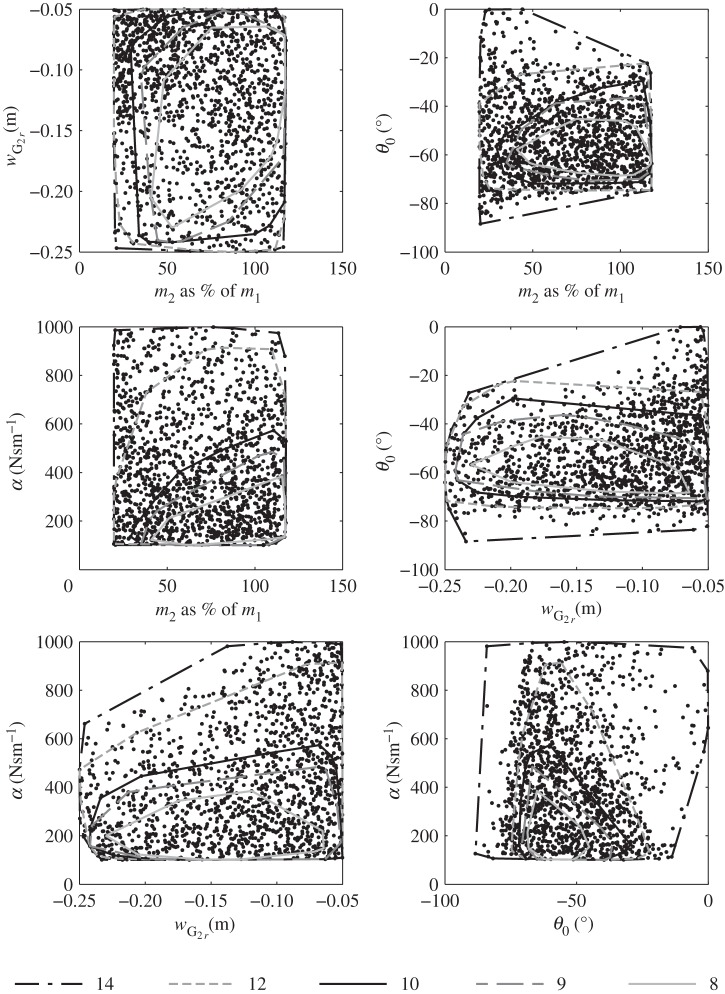
Experimental plan. Each dot represents one of the configurations from the dataset trimmed with a motion threshold of 14. The different lines correspond to the extent of the dataset trimmed by motion thresholds of 8, 9, 10, 12 and 14.

**Figure 2.**
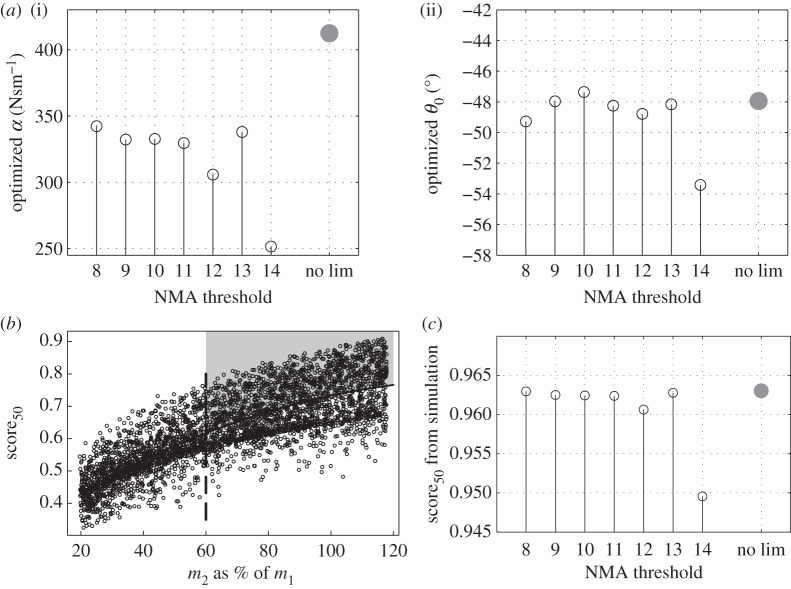
For (*a*,*c*), the black circles correspond to the optima from the dataset with the threshold ranging from 8 to 14. The grey dots correspond to the dataset trimmed as in (*b*). (*a*) Optimized PTO damping *α* (i) and angle *θ*_0_ (ii) as a function of the motion threshold used. (*b*) Experimental plan using 4000 configurations with no motion limit applied. The best configuration region retained for optimization is the top right quadrant (shaded area). (*c*) score_50_ calculated with the dataset obtained with a threshold of 14.

The threshold values selected for the study range from 8 to 14. With 8, only 81 configurations out of 4000 were kept, and the experimental plan is significantly reduced, as shown in figure 1. It was therefore not possible to select lower thresholds. Fourteen was used as an upper limit, yielding 1430 configurations. Finally, an optimization using the whole dataset, with no restriction on the normalized motion amplitude values, was conducted as a reference case. In this latter calculation, the experimental plan was limited to the top right quadrant of figure 2*b*, defined by the vertical boundary *m*_2_=0.6⋅*m*_1_ and the curve of equation ${\mathrm{score}}_{50}=a\cdot m_{2}^{2}+b\cdot m_{2}+c$, where *a*,*b* and *c* are defined by linear regression. The idea was to select the best configurations without altering the trend observed between score_50_ and the parameters (see [1] for details of the score_50_ calculation).

Optimal *m*_2_ and *w*_G_2___*r*_ appear to be independent from the motion threshold value, with *m*_2_=*m*_1_ and *w*_G_2___*r*_=−0.25 m, respectively . The influence of the threshold on the optimum PTO damping *α* and angle *θ*_0_ is presented in figure 2*a*(i,ii). Optimum *α* and *θ*_0_ vary relatively little with the NMA threshold, except when the latter is equal to 14. Even so, the overall range of optimum *θ*_0_, including that with no NMA limit, is small (between −46° and −54°). The optimum *α* range is larger but this needs to be kept in perspective because, as pointed out in the original study (§4c(iii)), the damping value has little effect on the optimization metric for *α*∈[200 500] Nms^−1^.

**Figure 3.**
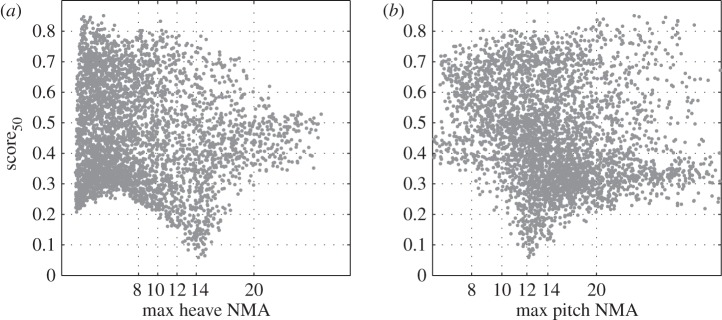
Distribution of score_50_ for the entire dataset as a function of the normalized motion amplitude maxima in heave (*a*) and in pitch (*b*).

Figure 2*c* shows the score_50_ of the optima for each threshold value. As for the optimum parameters, no significant variation is observed for thresholds from 8 to 13, as can be expected given that the optima *α* and *θ*_0_ are very similar. When using a threshold of 14, the score_50_ obtained is lower. It could appear as counterintuitive that relaxing the constraints on the dataset leads to a worse performing configuration. However, as can be seen in figure 3, high score_50_ values are not necessarily correlated with high NMA maxima, especially in heave. Increasing the NMA threshold therefore amounts to including in the dataset to be analysed a large number of low performing configurations. This can in turn make the optimization exercise more difficult. This is further supported by considering the optimization carried out with the ‘no motion limit’ (‘no lim’ in figure 2*c*) dataset whose performance is comparable to those of datasets associated with the tightest motion limits, but higher than the dataset with a threshold of 14.

Overall, this brief report shows that varying the NMA threshold value does not modify the fundamental conclusion of the original study, i.e. that there are clear advantages in using a sloped PTO, with an angle of approximately −50°. The metric selected for the WEC optimization in the original study is therefore robust. It provides optimal configurations that are to a large extent independent of the normalized amplitude response threshold.

## Data accessibility

This article is solely based on numerical modelling and therefore has no data associated with it.
